# Introducing “Research Matters”

**DOI:** 10.1371/journal.ppat.1005014

**Published:** 2015-06-25

**Authors:** Grant McFadden, Kasturi Haldar

**Affiliations:** 1 Department of Molecular Genetics and Molecular Biology, University of Florida, Gainesville, Florida, United States of America; 2 Boler-Parseghian Center for Rare and Neglected Diseases, University of Notre Dame, Notre Dame, Indiana, United States of America; 3 Department of Biological Sciences, University of Notre Dame, Notre Dame, Indiana, United States of America

In this issue of *PLOS Pathogens*, we are introducing a new front matter series to allow individual scientists from the many fields that encompass our community of editors, authors, and readers to comment on why the fundamental research in their labs, and that of their collaborators, matters. The genesis of this idea comes from the apparent gulf between working scientists and the general public that seems to be growing ever wider. In particular, there seems to be an expanding gap between what basic researchers and scientists try to accomplish in terms of scientific advancement, and what nonscientists, such as the lay public and the political world, perceive to be accomplished. This diminishes the deliverables expected as a result of funding basic research, the overall value of science to society, and the rational control of scientific funding. We seek this new Research Matters format for individual scientists to “say” in public how diverse fundamental research into pathogens assures real and compelling impact on public health, human knowledge, and life. Our goal is to evolve a forum for active scientists to speak directly, without filters or publicity agents, about why basic research in their field matters. Over time, we hope to develop a collective voice for our community while still preserving the authentic nature of the individual perspective.

To initiate this new series, we (GM and KH) have included our own specific examples. Our two essays have come from our own personalized viewpoints. We do not expect future pieces from others to copy our templates but rather to be used as a launching pad to communicate the amazing diversity of scientific advances that can come from fundamental curiosity-based research. If you are interested in contributing an essay for the series, please contact *PLOS Pathogens* at plospathogens.org to request specific guidelines that address content, structure, and length.

As this Research Matters series grows to include others engaged in the wider pathogen research enterprise, we expect these essays to form a library, the components of which might resonate with readers outside of our normal working groups, such as political and governmental policymakers, philanthropic leaders, disease-specific stakeholders and, of course, the general public. We hope to foster a wider consensus understanding that, in preparing for the unknown health issues of the future, the general public welfare is best served by fundamental research rather than politically reactive programs short on both memory and innovation.

